# Irish general practitioner (GP) perspectives on impact of direct access radiology on patient care in the community: results from a mixed-methods study

**DOI:** 10.1007/s11845-023-03419-1

**Published:** 2023-06-24

**Authors:** Michael Edmund O’Callaghan, Ronan Fawsitt, Jiaran Gao, John Broughan, Geoff McCombe, Amy Phelan, Diarmuid Quinlan, Claire Collins, Fintan Stanley, Walter Cullen

**Affiliations:** 1 Irish College of General Practitioners (ICGP), Dublin, Ireland; 2 School of Medicine, University of Limerick (UL), Dublin, Ireland; 3 Ireland East Hospital Group (IEHG) GP Research Network, University College Dublin/Ireland, Dublin, Ireland; 4 School of Medicine, University College Dublin (UCD), Dublin, Ireland

**Keywords:** Diagnostic imaging, General practice, Hospital avoidance, Primary care, Radiology, Referral

## Abstract

**Background:**

Since winter 2020/21, general practitioners (GPs) in the Republic of Ireland (RoI) have been granted access to diagnostic imaging studies on a new publicly funded pathway, expediting access to services previously obtained via hospital-based doctors.

**Aims:**

Outline GP perspectives on imaging studies obtained via the new “GP Access to Community Diagnostics” initiative.

**Methods:**

A mixed-methods design was employed. Referrals over the first six months of 2019 and 2021 were collated by a private imaging provider, and a randomly selected subset of 2021 studies (maximum 30 referrals per GP) was returned to participating GPs to provide detail on the impact on each patient’s care. In-depth qualitative interviews were also conducted with participating GPs.

**Results:**

Eleven GPs supplied detailed information on 81 studies organized through the new initiative. GPs reported that the initiative had led to a large proportion of cases being managed solely in general practice, with an 81% reduction in referrals to acute hospital settings and a 58% reduction in referrals to secondary care clinics. GPs felt imaging studies improved patient care in 86% of cases and increased GP workload in 58% of cases. GP qualitative interviews revealed four key themes: improved patient care, increased GP workload, reduction in hospital referrals, and opinions on ongoing management of such initiatives, including guidelines.

**Conclusions:**

GPs felt enhancing access to diagnostics improved patient care by expediting diagnosis, decision-making, and treatment and by reducing hospital referrals. GPs were generally positive about the initiative and made some suggestions on future management of the initiative.

## Introduction

Healthcare systems that manage health issues at the earliest opportunity in primary care are more efficient and cost effective [1]. Thus, initiatives that increase purposeful healthcare activity in our communities, thereby detecting issues “upstream” insofar as possible, must be closely examined.

Diagnostic imaging, a broad and growing field within the specialty of radiology, involves radiological technologies to visualize and image the internal structures of the body to rule out or detect disease and inform treatment plans [2, 3]. Historically, these technologies have been located in hospital settings and have been more accessible to hospital-based doctors, compared to general practitioners (GPs) [4–6].

Evidence indicates that enhancing GP access to diagnostics can lead to cost savings [7–10], appropriate use of imaging studies [11–16], improved patient outcomes [17, 18], and reductions in hospital referrals [19–21]. Other evidence points to overuse of imaging studies by GPs [22, 23]. Research to date tends not to include effects on GP workload as an outcome measure, instead concentrating on more hospital-focused metrics.

Increasing access to diagnostic imaging requires careful planning, given the need to rationalize health spending [24, 25], radiation concerns [26], potential for incidental findings [27], and the need to ensure access extends to socially disadvantaged groups [28]. Given the broad and complex range of matters to consider, narrowly focused approaches such as those examining direct cost of imaging may be superseded by more patient-centered and broader “value-based healthcare” metrics [29–34].

The Republic of Ireland (RoI) is somewhat unique in Europe in that 42% of the population qualify for free at point-of-care GP visits, while the remainder pay out-of-pocket for GP services [35]. In addition, 44% of Irish citizens have a private health insurance (PHI) policy [36], which frequently offer partial reimbursement for GP visits and timely access to hospital-based services, including diagnostic imaging. Public-only patients attending general practice traditionally have had extremely limited access to diagnostic imaging, particularly for more advanced modalities [37]. GPs have thus been required to refer many patients requiring certain studies (e.g., MRI brain) to a hospital-based doctor in an emergency department, rapid-access acute medical unit, or outpatient department to endorse and organize the same imaging study, resulting in significant delay, increased cost, and patient risk arising from delayed management. There can be little doubt that this situation has led to unnecessary waits, which is particularly regrettable when Irish public-only patients face some of the longest outpatient waiting times in Europe [38].

However, in recent years, there has been political support in RoI for the “Sláintecare” plan, which aims to move care currently delivered via hospitals into primary care settings [39]. Since late 2020, Irish GPs and their patients have been granted access to a new publicly funded diagnostic imaging pathway, the “GP Access to Community Diagnostics” initiative [40, 41]. This enables GPs to refer patients for X-ray, computed tomography (CT), magnetic resonance imaging (MRI), and dual-energy X-ray absorptiometry (DEXA) studies via private imaging providers. In March 2021, it was decided to retain these services solely for those adults entitled to free GP care [40], before this was again extended to full population coverage in May 2021. Over the course of 2022, over 250,000 imaging studies were funded by the initiative [42].

Given that population-level access to diagnostics is unprecedented in Irish healthcare, this project seeks to appraise GP views on diagnostic imaging in general, while specifically focusing on the impact of imaging provided via the new initiative.

## Methods

### Study design and population

This explorative study followed a mixed-methods design [43], which included a quantitative analysis of GP referrals for imaging studies, combined with 10–15-minute qualitative interviews of the same GPs.

The study was conducted with GPs from the University College Dublin (UCD)/Ireland East Hospital Group GP Research Network in collaboration with a private imaging provider, UCD School of Medicine and the ICGP Research Hub. All GPs (*n*=14) in this GP Research Network were invited to participate by email from UCD school of Medicine, with 11 GPs agreeing to take part.

### Ethical approval

Ethical approval was granted by the Irish College of General Practitioners (ICGP) Research Ethics committee on 28th October 2021 (ICGP_REC_21_0046).

### Quantitative evaluation: part i

Referral data from participating GPs held by the imaging provider were extracted and collated. All referrals from each GP for diagnostic imaging studies from January–June 2019 (pre-pathway introduction) to January–June 2021 (post-pathway introduction) were included.

### Quantitative evaluation: part ii

A random sample of maximum 30 referrals from the January–June 2021 period was sent to each GP to provide further information on each care encounter. GPs were asked the following questions for each of these 30 cases:

“If you didn’t have access to a scan/imaging study on the day, what would you have done?”

“What actually happened as a consequence of the report?”

“Did access to diagnostics alter care for this patient?”

“How has access to diagnostic imaging impacted your practice’s workload in the care of this patient?”

Analyses were carried out using R [44], and descriptive statistics were used to present findings.

### Qualitative evaluation

Participating GPs were also invited to partake in qualitative interviews via telephone. These interviews were carried out by a member of the research team (JG) by telephone during June and July 2022 and lasted between 10 and 15 minutes. A semistructured interview format was adopted to allow flexibility of response and follow-up for “unplanned” questions via a participant-led exploration of topics [45, 46]. All interviews were audio recorded and transcribed verbatim and checked for accuracy. All participants were given a code (e.g., P1), and thematic analysis was carried out using NVivo V12 software [47]. Key themes were determined by statements deemed important to the appraisal of use of diagnostic imaging in general practice. Similar concepts from the transcripts were identified and grouped together. Overarching themes were then identified and coded by examining the similarities and relationships between different concepts. Reliability was enhanced by two authors (JG and GM) independently analyzing the transcripts followed by a discussion of codes, themes, charted summaries, and interpretations to agree final themes.

## Results

### Quantitative data—part i

Over the first six months of 2019 (pre-period), the 11 participating GPs referred 223 patients to the imaging provider for 296 imaging studies (Table 1). Of these scans, 122 (41%) were funded by private health insurance (PHI), 87 (29%) funded by out-of-pocket payments, and additional private schemes (e.g., employer’s schemes and sports club schemes) were responsible for a further 86 (29%), with just one study funded by the HSE. There were 391 patients sent for 497 imaging studies in the corresponding 2021 period (post-period). Of these, the HSE GP Access to Community Diagnostics scheme accounted for 177 (36%) studies, PHI for 109 (22%), and out-of-pocket for 101 (20%), while other HSE schemes and private schemes were responsible for 58 (12%) and 52 (10%), respectively.

Regarding modalities, across all payer types and both study periods, there were 447 MRI studies, 154 DEXA studies, 109 X-ray studies, 82 USS, and 7 CT scans organized by the 11 GPs. Waiting times for all modalities increased across all schemes between early 2019 and early 2021, with median time from referral to scan increasing from nine days in January–June 2019 to 15 days in January–June 2021 (see Table 1).

**Table 1:**
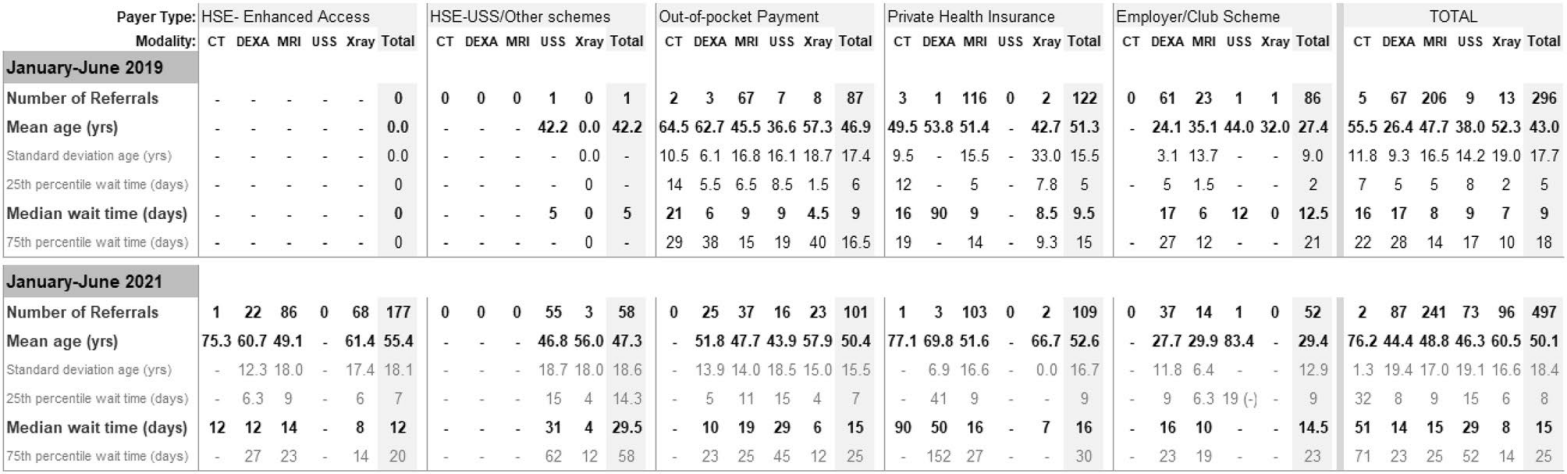
All GP referrals to the Private private Imaging imaging Provider provider during January to June 2019 and January to June 2021. Note HSE GP Direct Access to Diagnostics initiative was introduced in December 2020/January 2021

The  table 1 omits 4 studies from 2019 and 2 studies from 2021 where payer type was recorded as “unknown”

### Quantitative data—part ii

Participating GPs submitted more detailed information on 252 of the 497 referrals during the period January to June 2021 (post-period, see Table 2). Of these 252 referrals, 81 were funded by the HSE GP Access scheme, 73 by PHI, 51 by out-of-pocket payments, 34 by the HSE Ultrasound initiative, and 11 by private schemes.

Were imaging unavailable, GPs reported that 42 patients would have been sent to an ED/acute medical unit, which decreased to 5 cases when imaging was possible. With respect to onward outpatient department or consultant referral, without imaging GPs reported they would have referred 171 cases, which was reduced to 74 due to imaging availability. Large decreases in onward referrals reported by GPs resulted in large increases in patients’ ongoing management remaining in primary care. Indeed, GPs reported that presentations managed in general practice rose from 31 (12%) without imaging to 163 (65%) with timely access to imaging.

Of the HSE GP Access initiative studies, GPs reported that 16 out of 81 (20%) patients would have required urgent hospital assessment if imaging was not available. This number reduced to three following imaging. Regarding the need for referrals to outpatients or a consultant, the corresponding numbers were 55 without the scheme, decreasing to 23 with the scheme in place.

For PHI-funded studies, GPs reported that 10 of these patients would have required an acute floor referral were an imaging study pathway unavailable, which decreased to 1 with readily accessed PHI-funded scans. Fifty-three patients with PHI would have been sent for outpatient or consultant referral without imaging availability, and this decreased to 32 with accessible imaging.

As shown in Table 2, GPs were also asked whether referral for imaging altered patient care. GPs reported that access to all imaging modalities had a beneficial effect on patient care in at least four-fifths of presentations, with DEXA and MRI regarded being most beneficial of the commonly used modalities (i.e., CT excluded).

Finally, GPs were asked if referring for imaging altered practice workload. For all 252 studies, GPs reported that accessing imaging increased GP workload in 51% of cases, while there was no change in workload for 28% of studies and 21% of studies decreased workload. Corresponding figures for workload impact of the HSE GP Access to Community Diagnostics imaging studies specifically were 58% (increase), 28% (no change), and 14% (decrease).

### Qualitative data

Of the 11 participating GPs, 10 completed the qualitative interview. One GP was unable to complete the interview due to time pressures. Four key themes were identified from the analysis of the data:

1. Patient care

2. Practice workload

3. Hospital referrals

4. Management of the scheme

#### Theme one: patient care

All the GPs interviewed reported that direct access to services enhanced patient care at their practices.

*“It improved care for our patients. Because it has enabled us to make diagnoses quicker. It allowed us to manage more in the community and avoid referral to hospitals where waiting times are long”. p6*

*“It brings clarity to the diagnosis. It means patients don’t need to be referred to hospital unnecessarily for scans, and it means speedier care for the patient, follow-up tends to be speedier and better, resulting in what are probably better outcomes for patients because there isn't an extended delay in providing care.” p9*

GPs also stated that they felt direct access to diagnosis improved patient safety.

*“It’s a safety feature as well. It does provide extra safety for the patients, especially with regards to when there are prolonged delays when you're worried about patients’ health care risk.” p9*

GPs reported that enhanced access to diagnostics significantly reduced patient waiting time to access such services. This was deemed to lessen pressure, not only on GPs and their patients but also on hospital outpatient clinics.

“In general, most X rays are done within two or three weeks. MRI is within four weeks. If you send patients through the public system through the hospital, they’d be waiting six or up to nine months for an ultrasound. But if you send patients to Affidea* via this system, the waiting list is about 4 to 6 weeks, or maybe a little longer sometimes. But if the GP writes “urgent” on it is done much more quickly.” p1, *Affidea = private imaging provider

*“For the patients, they have much quicker access to that diagnosis. It could have taken one or two years for them to be seen before and have the scan, now the wait is only a matter of weeks and we can discuss it.” p7*

It was also highlighted that enhanced GP access to diagnostics had a positive impact in diverting patient care from the hospital system into the community.

*“So that culture of shifting care from hospitals into the community changed. The enhanced level of access to diagnostics is a vital part of that process (i.e., shifting care to the community) … So, in fact, this change will integrate care more effectively. General practice can now actually manage what it can manage properly, and only those patients who need the community hubs will need to be sent to them. So that’s using the system well.” p1*

GPs also reported that they felt patients were very satisfied with GPs having direct access to diagnostics.

*“For the patients, I think they’re generally pleased that they are now able to access investigations in a more timely manner.” p3*

*“I think the patients in general are very in favour of GPs being able to access scans, they prefer to have their problems dealt with by us locally than to go on to the hospitals.” p6*

#### Theme two: practice workload

GPs generally reported an increased workload, due to factors such as increased consultation rates, following up on results, contacting patients, and sharing decision-making around ongoing care.

*“It has increased the workload a little bit because you must take the time to refer them and to make sure the electronic referral is done. That’s fairly straightforward. Then the electronic X-Ray reports will come through some days, weeks later, and you must take the time to follow it up. You have to either call the patient, text the patient, bring the patient back in, review the results, and decide on if there should be any change in the care plan, so it does increase workload a little bit.” p1*

Some GPs detailed the context within which they work, as this initiative needs to find space in an already packed schedule. Most GPs pointed out that while there is an increased workload, enhanced GP access to diagnostics also brings significant benefits to their patients, such as large reductions in patient waiting times and better access to services, which also reduces hospital referrals. It may also create more efficient and timely management of patient issues, which may reduce workload in the long term.

*“You can’t put it in isolation; if we want to offer quality service then we need to have access to these tests. If you look at the overall healthcare system as an integrated system, primary, secondary, and community care, enhanced GP access to diagnostic imaging is very much saving time. You’re able to more appropriately refer and have a more effective outpatient if the patient goes to hospital with their diagnostics tests done in advance, and if they are going to the right place…that being to the right secondary care specialist. So, if the patient gets the answer more quickly, that leads to better patient satisfaction and better access to the service they need in a quicker way. Enhanced access has a lot of benefits for both clinicians and patients with regards to managing their problems.” p10*

#### Theme three: hospital referrals

All the GPs reported that direct access to diagnostic imaging reduced hospital referrals.

*“Direct access saves these patients being referred to hospital for their diagnostics, whether it’s MRI, ultrasound, X ray. Patients don’t have to be referred to a hospital acute floor, and indeed for them and for others, they don’t need to be put on a waiting list to be seen at the hospital to get diagnostics done. So, it’s made a huge difference in that area. It’s made a difference to patients as well as to doctors.” p1*

*“I think it certainly has reduced the number of patients that I’ve had to refer to hospitals and it certainly reduced the number of patients that I’ve had to refer to the emergency department.” p2*

Some GPs felt that the initiative would likely reduce outpatient referrals more than urgent emergency department or acute medical unit referrals.

*“I think that’s probably the biggest impact was on outpatient referrals rather than urgent referrals.” p7*

“It won’t stop all the casualty* referrals because the urgent things still need to go to casualty. ...For the less urgent routine outpatient scans, it does stop them from being referred into hospital.” p9, *casualty = ED

Other GPs outlined specific cases where urgent hospital referral was avoided.

*“I saw a man a few weeks ago who had a history of pancreatic cancer with weight loss and abnormal liver function tests. I would have had to refer him to the emergency department to get a scan urgently because it would not have been possible to arrange outpatients quickly enough. But with enhanced access to diagnostics I was able to arrange that scan myself, so he didn’t need to go to the emergency department.” p7*

#### Theme four: management of the scheme

GPs highlighted that the scheme is aligned with policy to manage more patient issues in the community.

*“The whole thrust of Sláintecare is to give General Practice and the community the wherewithal, the access to diagnostics, consultant advice, whatever is needed. It helps allied health professionals to manage patients in the community rather than referring everything to hospital. This enhanced access to diagnostics is a vital part of that process…” p1*

Most GPs acknowledged guidelines may be useful for rationalization of service use and to avoid wasting patient time and inappropriate radiation exposure, although not all agreed. However, some GPs pointed out that flexibility within guidelines would be important given the “gray areas” that exist in medical practice.

*“I think guidelines would be very useful. I think we need to make sure that if we’ve got access to diagnostics, that they're used appropriately in order to avoid wasting patients’ time and inappropriate exposure to radiation. We must appreciate that this is a valued resource which is finite, and we must use it in the most clinically efficient or cost efficient way. So, I think agreed guidelines that are accessible and jointly agreed on or pathways for use would be very important.” p10*

*“Guidelines are useful but they can be tricky too. What we want are guidelines that we can choose to use as appropriate, because every patient is unique, and every situation is unique. Guidelines should not be restricting access to diagnostics. Having guidelines should inform how you access diagnostics, but it shouldn’t limit it. I would not be in favour of overly bureaucratic guidelines. Medical doctors work with medical knowledge and exercise judgment. Guidelines are welcome, but they should not be restrictive.” p1*

*“I don’t think I’d like new guidelines. I think most doctors know the guidelines anyway because to refer patients you would have had to know the guidelines. I would hate to see guidelines come in because to me, that would probably reduce access even further.” p4*

## Discussion

### Main findings

This study of a sample of Irish GPs offers appraisal of a new publicly funded pathway which addresses previous deficiencies in access for those without PHI or the means to pay out-of-pocket for imaging studies. Though conducted on a small sample of GPs in a university-based research network, there was high participation from GPs invited and low attrition in completing all aspects of the study.

Participating GPs swiftly adopted the new HSE imaging pathway options, with a 68% increase in imaging referrals between the two study periods. Twenty percent of imaging studies in 2021 were paid for out-of-pocket (OOP) by patients, even though the entire population was eligible for the GP Access to Community Diagnostics initiative for four months of this period. The two-month period (March–April 2021) where population-wide coverage was removed, only to again be re-instated in May 2021 [48], likely impacted on GP use of the initiative. Interestingly, use of the GP Access to Community Diagnostics initiative to avail of plain film X-Ray and DEXA scans was commonplace, with these modalities comprising 51% of the total studies completed using the initiative. While these studies have traditionally been available to all patients through public hospitals, GP access to these modalities in public hospitals has been curtailed by the COVID-19 pandemic [41].

While retrospective review of patient cases, where imaging results are known, introduces potential for bias, GPs reported that access to imaging has considerable influence on how they manage patients. For example, for MRI imaging, GPs reported that 80 to 100% of cases (depending on the payer type, see Table 2) requiring an imaging study typically organized in an acute hospital setting avoided such a setting due to the timely availability of imaging. Regarding avoidance of an outpatient or consultant review, access to MRI stopped onwards referral in 37 to 73% of cases. These large reductions are partly explained by the prior system, where GPs in RoI could not access advanced imaging techniques for many patients. Influence of imaging on onward referral to nonacute care was the weakest for those patients with PHI, which may speak to the increased use of private medical care by this cohort [49].

GPs reported, in both quantitative and qualitative components, that access imaging increases GP workload in many cases. However, some GPs felt that patients accessing timely care at the community level may bring benefits for the broader health system.

It is not surprising that increasing GPs referrals to the imaging provider led to increased wait times. However, completion of three-quarters of scans within three weeks—moving to four weeks (increase of 33% in waiting time) despite a 68% increase in scan requests—represents a considerable improvement on previous pathways, where public-only patients faced lengthy waits for review prior to being referred for imaging.

**Table 2:**
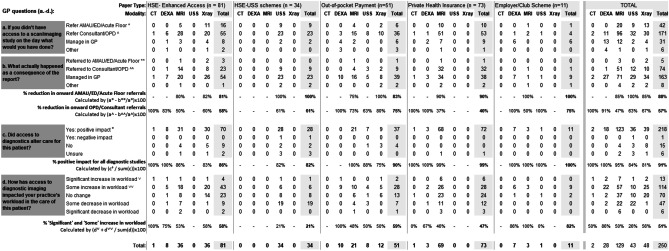
Subset of 252 GP referrals to the private imaging provider (selected at random) during January to June 2021. Note HSE GP Direct Access to Diagnostics initiative was introduced in late 2020. The table omits 2 studies where payer type was recorded as “unknown”

*AMAU* acute medical assessment unit, *ED* emergency department, *OPD* outpatient department

### Comparison with existing literature

As demonstrated elsewhere [19–21], our GP participants reported that access to diagnostic imaging reduced onward referrals to hospital considerably, regardless of how imaging studies are funded. More than four-fifths of cases requiring urgent imaging avoided acute referral when GPs had access to prompt imaging services. Referral to outpatient departments or secondary-care specialists was avoided for 75% of those who paid out-out-pocket, 58% of those sent for imaging on the HSE Enhanced Access to Diagnostics scheme, and 40% of those sent for imaging funded by private health insurance.

As seen in other studies [17, 18], participating GPs reported that imaging improved patient care in more than four-fifths of cases. These improvements were described in some detail during the qualitative interviews, with GPs reporting quicker diagnoses and more timely care as a consequence, which improved patient safety and reduced hospital referrals. Furthermore, GPs reported that patients themselves were pleased with the initiative and the ability to get imaging studies performed in a timely fashion while avoiding unnecessary hospital visits.

Participating GPs reported 51% of imaging studies increased GP workload—which has been shown in other studies [50, 51] and was one of this study’s main qualitative themes. To refer patients for imaging studies, GPs must meet patients, listen to their history, examine them, and then come to a decision with the patient regarding care with or without onward referral. GPs must also follow up on the results of imaging studies organized and convey findings to patients. On the other hand, accessing imaging in a timely fashion can expedite the investigation of a clinical problem such that it can be “closed out” and the patient reassured. Thus, GPs reported that while imaging usually increases their workload, there is potential for initiatives like this to both increase and decrease the work involved in dealing with a patient’s clinical problem(s).

The views of GPs in this project speak to the complexities involved in introducing guidelines for GPs in practice. In their interviews, participating GPs generally saw value in introduction of GP-appropriate guidelines to help guide continuation of this welcome initiative, but many felt guidelines should not be restrictive if introduced. Feedback from primary care clinicians reveals pressure from some patients, physician workload pressures, and lack of timely access to other options (e.g., physiotherapy) as barriers encountered when trying to apply guidelines in practice [52–54]. Direct engagement, and follow up engagement in the longer term, with feedback from both primary and secondary care seems important if guidelines are to work effectively [55, 56].

### Implications for future research

While results from this study reflect well overall on the introduction of GP direct access to imaging, the initiative represents a large investment in primary care nationally, and further in-depth studies, including cost-benefit analyses and patient views, should be carried out prospectively.

While barriers to utilization of guidelines in practice are described, there is a lack of studies focusing on the impact that diagnostic imaging has on general practice workload, particularly workload relating to those patients who have received an imaging study. While there are studies describing perceived overuse of imaging by primary care clinicians [22, 23], future studies in the area could usefully feature voices from both primary and secondary care to ensure a fair and shared consensus is reached. Establishing such consensus among clinicians is even more important as we seek to broaden appraisals to include patient views to assess the “value” of a diagnostic imaging intervention more holistically [29–34]. Primary care access to diagnostic imaging may benefit from additional interventions, such as audit and feedback for referring GPs [57] or interventions to show patients their own images [58, 59].

Aside from musculoskeletal issues, there are a lack of studies examining the specific clinical issues (and the various modalities that can help inform management) which may or may not be best managed within general practice. Further work in this area would help rank clinical issues and/or modalities to optimize effectiveness of combined GP and radiologist intervention in primary care. GPs referred just seven patients for CT scans over the study time period, perhaps due to concerns regarding radiation, the additional requirement for patients to have a recent kidney function result available (if intravenous contrast is required), or other reservations about use of this modality for patients being managed in general practice. Establishing an evidence base around primary care use of CT imaging will be important if this modality is to become part of modern general practice.

### Strengths and limitations

The mixed-methods approach facilitated the collection of comprehensive and rich data, thus simultaneously portraying the scale and complexity of the issue under investigation. Both sampling and methodology were felt to be appropriate and effective in undertaking an “early view” of the introduction of a new national care pathway for GP diagnostics.

Importantly, GP reporting was based on retrospective review of patient cases, where imaging results were known. This introduces potential for hindsight and social desirability bias. Nevertheless, GPs reported that existing pathways (e.g., imaging studies for patients with PHI and those paying out-of-pocket) and the new HSE GP Access to Community Diagnostics initiative were, in their opinion, similar in terms of effect on hospital avoidance. This may lead to the conclusion that the new initiative is at least having similar effects as existing pathways, which have been shown in other jurisdictions to lead to hospital avoidance [19–21].

The study is also limited by sample size, where our group may not capture the full breadth of GP opinions on the topic. Self-selection bias may be relevant for our participating GPs, both in terms of their participation in a GP research network and in this specific project. Lack of a control group further limits extensibility of findings. Effect on patient care was recorded from the GP’s perspective, following their review of each patient’s medical file. Objective assessment of “appropriateness” of referrals was not part of this study, partly due to a lack of standards against which to define this concept.

## Conclusion

GPs report that being enabled to access timely imaging studies for all patients in the RoI, regardless of their insurance cover or means, has had considerable impact on the management of many clinical scenarios in the community, which is consistent with the Sláintecare core objective of providing “the right care, in the right place, at the right time” [39]. The initiative also addresses fundamental issues regarding adequacy and equality of access for disadvantaged patient groups.

Our results indicate that improved access to diagnostics appears to have had benefits both for patients and the health service, by improving care provided in the community and reducing referrals to hospitals. The previously inequitable Irish system for community diagnostics where many necessary imaging studies were delayed due to rationing of essential and timely healthcare provides important context for this research area.

GPs reported that organizing and following up on imaging studies typically increased their immediate workload. While this study illustrates some differences between modalities, further work is required to examine the various clinical issues and their suitability for community-based management. Strategies to dissuade patients keen for onward referral for inappropriate imaging need to be built upon the bedrock of general practice, i.e., familiarity with our patients, continuity of care, and expertise in the gatekeeper role [59].

Modern general practice needs to remain wary of innovation for innovation’s sake [60–63], yet this initiative is a clear improvement on the historical disparity in access to imaging and definitive medical care previously inherent in our healthcare system. Ensuring commonality between community and hospital settings in terms of clinical indications for imaging studies, equity of access on a population basis, and guidelines to govern access for all patient groups are important considerations for the continued evolution of diagnostic imaging’s role in the Irish healthcare system.

## Data Availability

Study data is available on request. Please contact the corresponding author at mike.ocallaghan@icgp.ie

## References

[1] Starfield B, Shi L, Macinko J (2005) Contribution of primary care to health systems and health. Milbank Q 83(3):457–502. 10.1111/j.1468-0009.2005.00409.x. https://www.ncbi.nlm.nih.gov/pmc/articles/PMC2690145/10.1111/j.1468-0009.2005.00409.x PMC2690145 16202000

[2] Bercovich E, Javitt MC (2018) Medical imaging: from Roentgen to the digital revolution, and beyond. Rambam Maimonides Med J 9(4):e0034. 10.5041/RMMJ.10355 10.5041/RMMJ.10355 PMC6186003 30309440

[3] European Society of Radiology (2009) The future role of radiology in healthcare. Insights Imaging 1:2–11. 10.1007/s13244-009-0007-x 10.1007/s13244-009-0007-x PMC3259353 22347897

[4] Kamper SJ, Logan G, Copsey B et al (2020) What is usual care for low back pain? A systematic review of health care provided to patients with low back pain in family practice and emergency departments. Pain 161(4):694–702. 10.1097/j.pain.0000000000001751. (PMID: 31738226) 10.1097/j.pain.0000000000001751 31738226

[5] Downie A, Hancock M, Jenkins H et al (2020) How common is imaging for low back pain in primary and emergency care? Systematic review and meta-analysis of over 4 million imaging requests across 21 years. Br J Sports Med. 54(11):642–651. 10.1136/bjsports-2018-100087. (Epub 2019 Feb 13) 10.1136/bjsports-2018-100087 30760458

[6] Jensen MS, Olsen KR, Morsø L et al (2019) Does changed referral options affect the use of MRI for patients with low back pain? Evidence from a natural experiment using nationwide data. BMJ Open 9:e025921. 10.1136/bmjopen-2018-025921 10.1136/bmjopen-2018-025921 PMC6609080 31253612

[7] Appel CW, Balle AM, Krintel MM (2020). Direct-access to sonographic diagnosis of deep vein thrombosis in general practice: a descriptive cohort study. BMC Family Practice.

[8] Rua T, Mazumder A, Akande Y (2020). Management of chronic headache with referral from primary care to direct access to MRI compared with neurology services: an observational prospective study in London. BMJ Open.

[9] Pertile P, Poli A, Dominioni L et al (2015) Is chest X-ray screening for lung cancer in smokers cost-effective? Evidence from a population-based study in Italy. Cost Eff Resour Alloc 13(1) 10.1186/s12962-015-0041-0 PMC4567810 26366122

[10] DAMASK (Direct Access to Magnetic Resonance Imaging: Assessment for Suspect Knees) Trial Team (2008) Cost-effectiveness of magnetic resonance imaging of the knee for patients presenting in primary care. Br J Gen Pract 58(556):e10-6. 10.3399/bjgp08X342660. Erratum in: Br J Gen Pract 68(674):416. PMID: 19000394; PMCID: PMC2576309 10.3399/bjgp08X342660 PMC2576309 19000394

[11] Smith CF, Tompson AC, Jones N, Brewin J, Spencer EA, Bankhead CR, Hobbs FR, Nicholson BD (2018). Direct access cancer testing in primary care: a systematic review of use and clinical outcomes. British Journal of General Practice.

[12] Møller M, Juvik B, Olesen SC (2019). Diagnostic property of direct referral from general practitioners to contrast-enhanced thoracoabdominal CT in patients with serious but non-specific symptoms or signs of cancer: a retrospective cohort study on cancer prevalence after 12 months. BMJ open.

[13] de Schepper EI, Koes BW, Veldhuizen EF, Oei EH, Bierma-Zeinstra SM, Luijsterburg PA (2016). Prevalence of spinal pathology in patients presenting for lumbar MRI as referred from general practice. Fam Pract.

[14] Guldbrandt LM, Fenger-Grøn M, Folkersen BH et al (2013) Reduced specialist time with direct computed tomography for suspected lung cancer in primary care. Dan Med J 60(12) 24355447

[15] Guldbrandt LM, Rasmussen TR, Rasmussen F, Vedsted P (2014). Implementing direct access to low-dose computed tomography in general practice—method, adaption and outcome. PLoS One.

[16] Hughes P, Beddy P, Sheehy N (2015). Open-access ultrasound referrals from general practice. Ir Med J.

[17] DAMASK (Direct Access to Magnetic Resonance Imaging: Assessment for Suspect Knees) Trial Team (2008) Effectiveness of GP access to magnetic resonance imaging of the knee: a randomised trial. Br J Gen Pract 58(556):e1-8; discussion 774. 10.3399/bjgp08X342651. PMID: 19000393; PMCID: PMC2576308 10.3399/bjgp08X342651 PMC2576308 19000393

[18] Smith CF (2018). Direct access cancer testing in primary care: a systematic review of use and clinical outcomes. British Journal of General Practice.

[19] Rutten MH, Marleen Smits M, Peters YAS, Jan Assendelft WJJ, Westert GP, Giesen PHJ (2018). Effects of access to radiology in out-of-hours primary care in the Netherlands: a prospective observational study. Family Practice.

[20] Speets AM, Hoes AW, van der Graaf Y et al (2006) Upper abdominal ultrasound in general practice: indications, diagnostic yield and consequences for patient management. Fam Pract 23(5):507–11. 10.1093/fampra/cml027. (Epub 2006 Jun 21) 10.1093/fampra/cml027 16790453

[21] Berg HF, Vermeulen M, Algra PR, Boonman-de Winter LJ (2016) Direct access to magnetic resonance imaging improved orthopaedic knee referrals in the Netherlands. Fam Pract. 33(5):482–7. 10.1093/fampra/cmw035. (Epub 2016 May 26 PMID: 27230743) 10.1093/fampra/cmw035 27230743

[22] Karel YHJ, Verkerk K, Endenburg S et al (2015) ‘Effect of routine diagnostic imaging for patients with musculoskeletal disorders: a meta-analysis’. Eur J Intern Med 26(8):585–595. Available: 10.1016/j.ejim.2015.06.018. 10.1016/j.ejim.2015.06.018 26186812

[23] Sajid IM, Parkunan A, Frost K (2021) Unintended consequences: quantifying the benefits, iatrogenic harms and downstream cascade costs of musculoskeletal MRI in UK primary care. BMJ Open Quality 10:e001287. 10.1136/bmjoq-2020-001287 10.1136/bmjoq-2020-001287 PMC8256731 34215659

[24] Watt T, Charlesworth A, Gershlick B (2019) Health and care spending and its value, past, present and future. Future Health 6(2):99–105; 10.7861/futurehosp.6-2-99 10.7861/futurehosp.6-2-99 PMC6616184 31363514

[25] Health expenditure per capital OECD Health Statistics (2021) OECD website. Available at: https://www.oecd-ilibrary.org/sites/154e8143-en/index.html?itemId=/content/component/154e8143-en. (Accessed 31/8/22)

[26] Trapp JV, Kron T, editors (2008) An introduction to radiation protection in medicine. CRC Press

[27] Lumbreras B, Donat L, Hernández-Aguado I (2010). Incidental findings in imaging diagnostic tests: a systematic review. Br J Radiol..

[28] Karia A, Zamani R, Akrami M (2021). Socio-economic disparities in access to diagnostic neuroimaging services in the United Kingdom: a systematic review. Int J Environ Res Public Health..

[29] van Beek EJR, Kuhl C, Anzai Y et al (2019) Value of MRI in medicine: more than just another test? J Magn Reson Imaging 49(7):e14–e25. 10.1002/jmri.26211. (Epub 2018 Aug 25) 10.1002/jmri.26211 PMC7036752 30145852

[30] Push towards changing from “cost-based to value-based diagnostic algorithms with patient/pathology-tailored protocols which could ultimately provide major economical and medical benefits” ref: Ruivo C and Roriz D. (2019) ‘Value-Based Radiology in MSK Imaging’, in Value-Based Radiology, Cham: Springer International Publishing, 117–124. Available: 10.1007/174_2019_210.

[31] Thompson MJ, Suchsland MZ, Hardy V et al (2021) Patient-centred outcomes of imaging tests: recommendations for patients, clinicians and researchers. BMJ Quality & Safety Published Online First: 06 October 2021. 10.1136/bmjqs-2021-013311 10.1136/bmjqs-2021-013311 PMC10447372 34615733

[32] Brady AP, Bello JA, Derchi LE, Fuchsjäger M, Goergen S, Krestin GP, Lee EJ, Levin DC, Pressacco J, Rao VM, Slavotinek J, Visser JJ, Walker RE, Brink JA (2021). Radiology in the era of value-based healthcare: a multi-society expert statement from the ACR, CAR, ESR, IS3R, RANZCR and RSNA. J Med Imaging Radiat Oncol.

[33] Brady A, Brink J, Slavotinek J (2020). Radiology and value-based health care. JAMA..

[34] European Society of Radiology (ESR). Insights imaging (2021) 12:6. 10.1186/s13244-020-00943-x 10.1186/s13244-020-00943-x PMC7790952 33411144

[35] Department of Health (DOH), Ireland (2021) Report: “health in Ireland: key trends 2021”. Nov 2021. Available at: https://www.gov.ie/en/publication/350b7-health-in-ireland-key-trends-2021/. (Accessed 31/08/2022)

[36] The Health Insurance Authority, Ireland (2020) Health insurance market report for 2020. Available at: https://www.hia.ie/sites/default/files/Market%20Report_.pdf. (Accessed 02/10/2022)

[37] O'Riordan M, Doran G, Collins C (2015) Access to diagnostics in primary care and the impact on a primary care led health service 108 (2):53-5 Ir Med J. https://www.lenus.ie/handle/10147/558773. 25803958

[38] Björnberg A, Phang AY (2019) Health Consumer Powerhouse. Euro health consumer index 2018 report. Available at: https://healthpowerhouse.com/media/EHCI-2018/EHCI-2018-report.pdf. (Accessed 31/08/2022)

[39] Houses of the Oireachtas, Ireland (2017) Committee on the Future of Healthcare, Sláintecare report. Available at: https://assets.gov.ie/22609/e68786c13e1b4d7daca89b495c506bb8.pdf. (Accessed 12/10/2022)

[40] Health Service Executive (HSE), Ireland (2021) HSE website- GP diagnostics. January 2021. Available at: https://www.hse.ie/eng/services/list/2/primarycare/community-healthcare-networks/gp-diagnostics/. (Accessed 31/08/2022)

[41] Health Service Executive (HSE), Ireland (2020) HSE report “winter planning within the COVID-19 pandemic. October 2020 – April 2021”. Available at: https://www.hse.ie/eng/services/publications/winter-planning-within-the-covid19-pandemic-october-2020-april-2021.pdf. Accessed 31/08/2022)

[42] Health Service Executive (HSE) (2022) Enhanced Community Care Conference 2022 Dublin Castle. Session 1: Community Healthcare Networks (CHNs). Available at: https://www.hse.ie/eng/services/list/2/primarycare/enhanced-community-care/session-1-community-health-networks.pdf. (Accessed 02/10/2022)

[43] Fetters MD, Curry LA, Creswell JW (2013) Achieving integration in mixed methods designs-principles and practices. Health Serv Res 48(6 Pt 2):2134-56. 10.1111/1475-6773.12117. Epub 2013 Oct 23. PMID: 24279835; PMCID: PMC4097839. 10.1111/1475-6773.12117 PMC4097839 24279835

[44] R Core Team (2014) R: A language and environment for statistical computing. R Foundation for Statistical Computing, Vienna, Austria. URL http://www.R-project.org/

[45] DeJonckheere M, Vaughn LM (2019) Semistructured interviewing in primary care research: a balance of relationship and rigour. Family medicine and community health 7(2) 10.1136/fmch-2018-000057 PMC6910737 32148704

[46] Goodenough A, Waite S (2012) Real world research: a resource for users of social research methods in applied settings: Taylor & Francis

[47] Braun V, Clarke V (2014) What can “thematic analysis” offer health and wellbeing researchers?: Taylor & Francis 10.3402/qhw.v9.26152 PMC4201665 25326092

[48] Health Service Executive (HSE) website- Direct access to scans helps GPs keep patients out of hospital. 7th Dec 2021. Available at: https://www.hse.ie/eng/about/our-health-service/making-it-better/direct-access-to-scans-helps-gps-keep-patients-out-of-hospital.html. (Accessed 02/10/2022)

[49] Kapur K (2020) Private health insurance in Ireland: trends and determinants. The Economic and Social Review, Vol. 51, No. 1, Spring 2020, pp. 63–92. Available at: https://www.esr.ie/article/view/1386. (Accessed 25/5/23).

[50] Community diagnostic centres: bringing diagnostics closer to home (2021) Samuel WD Merriel, Lennard Lee, Richard Neal. Br J Gen Pract 71 (713):534–535. 10.3399/bjgp21X717701 10.3399/bjgp21X717701 PMC8686421 34824064

[51] Sajid IM, Frost K, Paul AK (2022). ‘Diagnostic downshift’: clinical and system consequences of extrapolating secondary care testing tactics to primary care. BMJ Evidence-Based Medicine.

[52] Hall AM, Scurrey SR, Pike AE, Albury C, Richmond HL, Matthews J, Toomey E, Hayden JA, Etchegary H (2019). Physician-reported barriers to using evidence-based recommendations for low back pain in clinical practice: a systematic review and synthesis of qualitative studies using the theoretical domains framework. Implement Sci..

[53] Pike A, Patey A, Lawrence R et al (2022) De-implementing Wisely Research Group, Hall A. Barriers to following imaging guidelines for the treatment and management of patients with low-back pain in primary care: a qualitative assessment guided by the Theoretical Domains Framework. BMC Prim Care 23(1):143. 10.1186/s12875-022-01751-6. PMID: 35659251; PMCID: PMC9164352 10.1186/s12875-022-01751-6 PMC9164352 35659251

[54] Matowe L, Ramsay CR, Grimshaw JM et al (2002) Effects of mailed dissemination of the Royal College of Radiologists’ guidelines on general practitioner referrals for radiography: a time series analysis. Clin Radiol 57(7):575–578, ISSN 0009-9260. 10.1053/crad.2001.0894. 10.1053/crad.2001.0894 12096854

[55] Hollingworth W, Todd CJ, King H et al (2002) Primary care referrals for lumbar spine radiography: diagnostic yield and clinical guidelines. Br J Gen Pract 52(479):475–80. PMID: 12051212; PMCID: PMC1314323 PMC1314323 12051212

[56] Tahvonen P, Oikarinen H, Tervonen O (2020) The effect of interventions on appropriate use of lumbar spine radiograph and CT examinations in young adults and children: a three-year follow-up. Acta Radiol. 61(8):1042–1049. 10.1177/0284185119893091. (Epub 2019 Dec 22 PMID: 31865752) 10.1177/0284185119893091 31865752

[57] O'Connor DA, Glasziou P, Maher CG, McCaffery KJ, Schram D, Maguire B, Ma R, Billot L, Gorelik A, Traeger AC, Albarqouni L, Checketts J, Vyas P, Clark B, Buchbinder R (2022). Effect of an individualized audit and feedback intervention on rates of musculoskeletal diagnostic imaging requests by Australian general practitioners: a randomized clinical trial. JAMA..

[58] Carlin LE, Smith HE, Henwood F (2014) To see or not to see: a qualitative interview study of patients’ views on their own diagnostic images. BMJ Open 4:e004999. 10.1136/bmjopen-2014-004999 10.1136/bmjopen-2014-004999 PMC4120403 25082418

[59] Walderhaug KE, Nyquist MK, Mjølstad BP (2022). GP strategies to avoid imaging overuse. A qualitative study in Norwegian general practice, Scandinavian Journal of Primary Health Care.

[60] Zigman Suchsland ML, Hardy V, Zhang Y et al (2019) Provider perspectives of patient experiences in primary care imaging. J Am Board Fam Med 32(3):392–397. 10.3122/jabfm.2019.03.18028857 10.3122/jabfm.2019.03.180288 PMC7050574 31068403

[61] Müskens JLJM, Kool RB, van Dulmen SA (2022). Overuse of diagnostic testing in healthcare: a systematic review. BMJ Quality & Safety.

[62] Cherryman G (2006) Imaging in primary care. Br J Gen Pract 56(529):563–4. PMID: 16882370; PMCID: PMC1874516 PMC1874516 16882370

[63] Chambers D, Booth A, Baxter SK et al (2015) Evidence for models of diagnostic service provision in the community: literature mapping exercise and focused rapid reviews. Southampton (UK): NIHR Journals Library. PMID: 28005317 28005317

